# The Effect of Methylphenidate and Atomoxetine on Heart Rate and Systolic Blood Pressure in Young People and Adults with Attention-Deficit Hyperactivity Disorder (ADHD): Systematic Review, Meta-Analysis, and Meta-Regression

**DOI:** 10.3390/ijerph15081789

**Published:** 2018-08-20

**Authors:** Edwin F. Liang, Samuel Z. Lim, Wilson W. Tam, Cyrus S. Ho, Melvyn W. Zhang, Roger S. McIntyre, Roger C. Ho

**Affiliations:** 1 Department of Psychological Medicine, Yong Loo Lin School of Medicine, National University of Singapore, Singapore 119228, Singapore; a0105307@u.nus.edu (E.F.L.); a0105316@u.nus.edu (S.Z.L.); su_hui_ho@nuhs.edu.sg (C.S.H.); 2 Alice Lee Centre for Nursing Studies, Yong Loo Lin School of Medicine, National University of Singapore, Singapore 119077, Singapore; nurtwsw@nus.edu.sg; 3 National Addiction Management Service, Institute of Mental Health, Singapore 539747, Singapore; melvynzhangweibin@gmail.com; 4 Mood Disorders Psychopharmacology Unit, University Health Network, Toronto, ON M5T 1R8, Canada; Roger.McIntyre@uhn.ca; 5 Institute of Medical Science, University of Toronto, Toronto, ON M5T 1R8, Canada; 6 Department of Psychiatry, University of Toronto, Toronto, ON M5T 1R8, Canada; 7 Department of Pharmacology, University of Toronto, Toronto, ON M5T 1R8, Canada

**Keywords:** methylphenidate, atomoxetine, cardiovascular system, heart rate, systolic blood pressure, meta-analysis, children, adults

## Abstract

*Objectives*: This meta-analysis aims to study the effects of atomoxetine and methylphenidate on heart rate (HR), systolic blood pressure (SBP), and a number of adverse cardiac events on patients receiving treatment for attention-deficit hyperactive disorder (ADHD) in comparison to placebo and between atomoxetine and methylphenidate. *Methods*: We searched the following databases: PubMed, EMBASE, and ScienceDirect. Meta-analysis was performed on studies that examined the relationships between methylphenidate or atomoxetine and HR, SBP, as well as a number of adverse cardiac events. These studies were either placebo-controlled or comparison studies between methylphenidate and atomoxetine. Meta-regression identified patient- and treatment-related factors that may contribute to heterogeneity. *Results*: Twenty-two studies were included and the total number of participants was 46,107. Children/adolescents and adults treated with methylphenidate had more significant increases in post- vs. pre-treatment HR (*p* < 0.001) and SBP (*p* < 0.001) than those treated by placebo. Children and adolescents treated with atomoxetine had more significant increases post- vs. pre-treatment HR (*p* = 0.025) and SBP (*p* < 0.001) than those treated with methylphenidate. Meta-regression revealed mean age of participants, mean dose, and duration of atomoxetine and methylphenidate as significant moderators that explained heterogeneity. There were no differences in the number of adverse cardiac events between participants with methylphenidate treatment and placebo or atomoxetine. *Conclusions*: Children/adolescents and adults treated with methylphenidate resulted in significant increases in post- vs. pre-treatment HR and SBP as compared to placebo. Similarly, children and adolescents treated with atomoxetine had significant increases in post- vs. pre-treatment HR and SBP than those treated with methylphenidate. These findings have potential implications for continuous monitoring of HR and SBP throughout the course of treatment although the risk for adverse cardiac events were insignificant.

## 1. Introduction

Attention deficit hyperactive disorder (ADHD) is a psychiatric disorder characterized by inattention, hyperactivity, and impulsivity. ADHD is common in children and adolescents [1]. In the United States, the prevalence ADHD among children 4–17 years of age had increased by 22% between 2003 and 2007, from 7.8% to 9.5% [2]. ADHD affects boys approximately three times more than in girls [3]. In general, ADHD symptoms persist to adulthood in 25%–50% of patients with childhood onset, while the prevalence of ADHD in adults is 2%–5% [4].

The Multimodal Treatment Study of Children and Adolescents found that medications with or without behavioural treatment is the most effective for core ADHD symptoms [5]. Recent updates by the National Institute of Clinical Excellence (NICE) recommend that methylphenidate is effective for children and young people with ADHD as well as ADHD and coexisting intellectual disability or substance abuse [6]. Methylphenidate acts by blocking the dopamine and noradrenaline transporters and increasing presynaptic release of dopamine and noradrenaline [7]. The NICE guidelines recommend atomoxetine if methylphenidate has been ineffective at the maximum tolerated dose, or if the patient is intolerant to methylphenidate. In contrast, atomoxetine is a noradrenaline reuptake inhibitor [8].

There is substantial concern from clinicians, patients, parents, and the public about the cardiovascular safety of ADHD medications [9]. The concern about the cardiovascular safety of methylphenidate was first reported in 1958 [10]. In 1976, blood pressure (BP) and heart rate (HR) were found to be increased significantly with methylphenidate therapy [11]. In 2012, it was found that children with ADHD have autonomic dysfunction [12]. Atomoxetine and methylphenidate therapy might further increase the cardiovascular risk. Lamberti et al. (2015) found that children treated by immediate-release methylphenidate had mean HR increased from 80.5 + 15.5 bpm to 87.7 + 18.8 bpm but no significant changes in ECG parameters [7]. In contrast, Ariceri et al. (2012) found that treatment with methylphenidate and atomoxetine in young people caused reduction in BP and HR after 24 months [13]. As a result, the effect of methylphenidate and atomoxetine on HR and BP remain inconclusive.

A 2012 systematic review reported mixed findings on the association between prescription of stimulants and adverse cardiovascular outcomes [14]. A 2014 systematic review reported that most of the studies did not yield statistically significant results for BP and HR in patients taking methylphenidate and atomoxetine but did not study factors that could affect BP and HR [15]. Mick et al. (2012) conducted a meta-analysis and found that adults with ADHD treated with stimulant medication showed increased BP and HR [16]. This meta-analysis did not study the effects of nonstimulant medication on HR and BP. Recently, Hennissen et al. (2017) published a meta-analysis to study cardiovascular effects of stimulant and nonstimulant medication for children and adolescents and found that both atomoxetine and methylphenidate caused significant post- vs. pre- increases in systolic blood pressure (SBP) and HR [17]. The head-to-head comparison of atomoxetine and methylphenidate did not show significant differences in SBP and HR. This meta-analysis was mainly based on open-label studies and lack of placebo groups. Further, this meta-analysis did not include adult participants and the cardiovascular safety of methylphenidate should be considered in adult patients [18]. Schelleman et al. (2012) could not find a causal association between methylphenidate and risk of serious cardiovascular events in adults [19]. The prevalence of cardiovascular adverse effects in adults is less commonly studied in the literature. The aforementioned meta-analyses did not report heterogeneity and identify moderators that explained heterogeneity. Cortese et al. (2018) performed a network meta-analysis to compare the efficacy and tolerability of different medications for treating ADHD [20]. This network meta-analysis did not measure the specific impact of methylphenidate and atomoxetine on blood pressure and heart rate in young people and adults. As a result, a new meta-analysis and meta-regression analysis to study factors that could influence SBP and HR is required.

For cardiovascular adverse effects, a nationwide self-controlled case series study was conducted in Korea. This study found that the relative risk of myocardial infarction and arrhythmia was increased after the start of methylphenidate treatment for ADHD in children and young people but not for hypertension, ischaemic stroke, and heart failure [21]. The Korean dataset was large but findings might not be generalisable to other countries. Olfson et al. (2012) concluded that cardiovascular events and symptoms were rare and not associated with stimulant use in young people [22]. This study did not explore asymptomatic changes in cardiovascular parameters. A systematic review suggested that six out of seven studies in children and adolescents did not show an association between stimulant use and adverse cardiovascular outcomes [14]. These preliminary findings require further confirmation.

Further, direct comparisons of the prevalence of cardiovascular adverse effects between methylphenidate and atomoxetine have not been performed to date. It is possible that certain risk factors are associated with higher risk of cardiovascular adverse effects. Having information on these risk factors will be helpful for healthcare professionals to identify patients who are more likely to develop cardiovascular adverse effects and apply necessary precautions. There has been a growing interest of cardiovascular safety of methylphenidate and atomoxetine in treating with patients suffering from ADHD. Additionally, examination of the effect of methylphenidate and atomoxetine on adults has yet to be completed, and studies that include adult patients with ADHD are generally small in this area of research. Hence, a meta-analysis is urgently needed to provide a better estimate of effect. The main objective of this meta-analysis was to conduct a head-to-head comparison of post- vs. pre-treatment HR, SBP, and the prevalence of cardiovascular adverse effects between patients taking methylphenidate and placebo or atomoxetine. This meta-analysis focused on SBP because SBP has been a better predictor of cardiovascular risk [23]. The second objective was to identify factors that are associated with higher HR, SBP, and risk of cardiovascular adverse effects.

## 2. Materials and Methods

### 2.1. Search Strategy

This meta-analysis adhered to a priori designed protocol. We searched the following databases: PubMed, EMBASE, and Science Direct. Our search started from inception of databases to 31 May 2016 for relevant articles. Our search terms were “methylphenidate” with varying combinations of other search terms: “atomoxetine”, “cardiovascular”, “heart rate”, “blood pressure”, “cardiovascular diseases”, “cerebrovascular accident”, “myocardial infarction”, “electrocardiography”, “vasculopathy”, “sudden cardiac”, and “death”. All fields were checked under the database search and suggested articles by databases were considered.

### 2.2. Inclusion Criteria

We included all trials published in the English language that compared methylphenidate to either a placebo or atomoxetine in terms of its cardiovascular safety in the context of treatment for ADHD. The trials included in the data analyses met the following inclusion criteria: (i) compare methylphenidate and either placebo or atomoxetine in ADHD treatment; (ii) include outcome data on HR, SBP, or number of cardiovascular adverse events during treatment; and (iii) no concomitant administration of other psychotropic medication during the trial.

We screened all citations and abstracts from the search strategy and identified articles for full-text extraction. Two investigators (ELC and SLZ) performed the literature search, screening, and data extraction independently. Disagreements at any phase of the review process were resolved by discussion. If a consensus was not reached, a third independent rate (RCH) determined eligibility. Studies that met the following exclusion criteria were excluded: (i) without a placebo control arm; (ii) methylphenidate or atomoxetine not used in the trial arm; (iii) nonhuman studies; (iv) inadequate sample size (less than 5); (v) review articles; and (vi) non-English articles and English abstracts that could not provide adequate information to calculate effect size. All data were independently extracted and organized into a standard electronic data extraction form. All publications were reviewed as full texts.

### 2.3. Outcome Measures of This Meta-Analysis

The primary outcomes were the proportion of patients who experienced changes in cardiovascular parameters (i.e., post- vs. pre-treatment HR and SBP) after methylphenidate or atomoxetine treatment. Secondary outcomes include the occurrence of adverse cardiac outcomes, namely cerebrovascular accidents, myocardial infarction, sudden cardiac death, and significant electrocardiogram changes.

### 2.4. Assessment of Quality of Trial

The quality of each trial was independently assessed according to the standard Jadad scoring system [24]. The assessment was based on: (i) whether the randomization method was appropriate; (ii) whether double blindness was mentioned in the trial and whether it was appropriately performed; and (iii) whether the number of patients who withdrew and dropped-out of the study and their respective reasons were clearly stated. The Jadad score ranges from 0 to 5, with higher scores indicating better quality of the trial. The calculated mean Jadad scores are presented in Table 1.

### 2.5. Statistical Analysis

This meta-analysis was reported according to the Preferred Reporting Items for Systematic Review and Meta-Analyses (PRISMA) guidelines [25]. All statistical analyses were performed using Comprehensive meta-analysis. This meta-analysis used a random-effects model that assumed heterogeneity between studies and their respective effect sizes [26,27]. We used standardized mean difference to establish the overall effect size of the difference in post- vs. pre-treatment HR and SBP as well as a number of adverse cardiac events between the pharmacological treatment (i.e., methylphenidate or atomoxetine) and placebo or between two pharmacological treatment (i.e., methylphenidate vs. atomoxetine) in each of the studies and presented our findings in the forest plots. We reported the results using 95% confidence interval (CI). We performed subgroup analysis by comparing children/adolescents and adults. Between-group effect was reported and a *p* value of <0.05 was taken as significant. Between-study heterogeneity was assessed with the I^2^ statistic [28]. As a guide, I^2^ values of 25% were considered low, 50% moderate, and 75% high [29]. For models with considerable heterogeneity, a meta-regression was performed to identify the moderators which might contribute to the heterogeneity of the effect sizes if there were at least four studies included in the meta-analysis [30]. The regression coefficients (β) and the associated z values and *p* values were reported in the meta-regression analysis. Egger’s test was performed to assess for presence of publication bias. In the event that publication bias was detected, the classic fail-safe test was performed to establish the potential number of missing studies [31].

## 3. Results

### 3.1. Articles Included in Data Analyses

**Figure 1:**
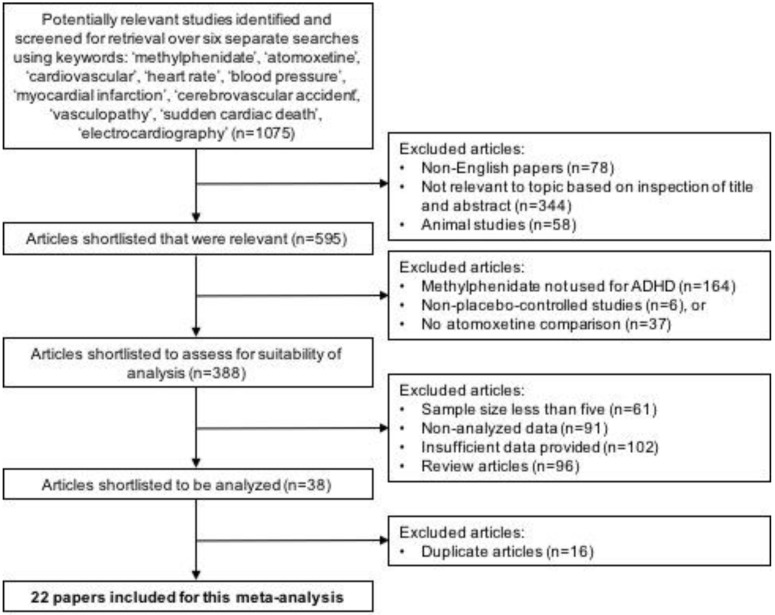
Preferred Reporting Items for Systematic Review and Meta-Analyses (PRISMA) flowchart summarizing results of literature search.

Out of the 1075 potentially relevant articles identified in our initial searches, a total of 22 articles were included in our analysis. Studies were excluded at each stage of screening for inclusion and exclusion criteria (see Figure 1). Out of the 22 studies, 18 studies were randomized controlled trials (RCTs), 2 studies were cohort studies, and 2 studies were retrospective cohort studies. A detailed description of study characteristics of included studies is presented in Table 1. Fourteen studies compared the effect on post- vs. pre-treatment HR and SBP between methylphenidate and placebo. Eight studies compared the effect compared the effect on post- vs. pre-treatment HR and SBP between methylphenidate and atomoxetine. In total, 39,996 patients received methylphenidate treatment, 5274 patients received atomoxetine treatment, and 837 patients received placebo. Nine studies recruited children participants only, six studies recruited children and adolescents, and seven studies trials recruited adult participants. The study period ranges from 1977 to 2015. Ten studies were published in the last 10 years.

### 3.2. Heart Rate

#### 3.2.1. Comparing Post- vs. Pre-Treatment HR between the Methylphenidate and Placebo Groups (Children/Adolescents and Adults)

**Figure 2:**
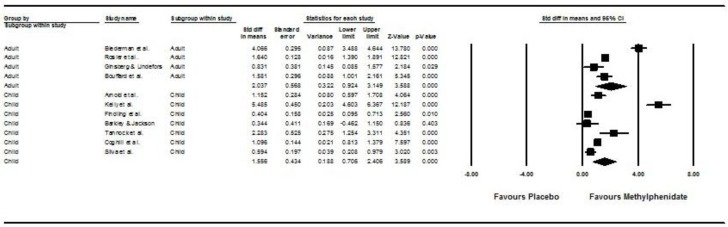
Forest plot and subgroup analysis on post- versus pre-treatment heart rate (HR) between placebo and methylphenidate groups.

Figure 2 shows the results of the 11 studies that compared the pre- and post-treatment HR between participants taking methylphenidate and placebos [32,33,34,35,36,37,38,39,40,41,42]. Children and adolescents treated with methylphenidate had a more significant increase in post- vs. pre-treatment HR than those treated by placebo (pooled standardized mean difference (SMD) with random-effects model: 1.56, 95% CI: 0.71–2.41, z = 3.59, *p* < 0.001). Adults treated with methylphenidate had a more significant increase in post- vs. pre-treatment HR than those treated by placebo (pooled SMD with random-effects model: 2.04, 95% CI: 0.92–3.15, z = 3.59, *p* < 0.001). Subgroup analysis showed no significant difference between children/adolescents and adults in post- vs. pre-treatment HR (Q = 0.45, *p* = 0.5). A significant level of between-study heterogeneity was found (τ^2^ = 1.20, Q = 236.66, df = 10, *p* < 0.001, I^2^ = 96.197). When we undertook meta-regression to explore the impact of our a priori sources of heterogeneity (Table 2), we found significant effects of mean age of participants (β = 0.0032, z = 7.31, *p* < 0.001), proportion of male gender (B = −1.88, z = −4.5, *p* < 0.001), duration of treatment (B = 0.011, z = 2.06, *p* = 0.04), and mean dose of methylphenidate (β = 0.032, z = 6.53, *p* < 0.001). There was no publication bias (intercept = 4.46, SE = 3.98, df = 8, *p* = 0.29).

#### 3.2.2. Comparing Post- vs. Pre-Treatment HR between the Methylphenidate and Atomoxetine Groups (Children/Adolescents Only)

**Figure 3:**
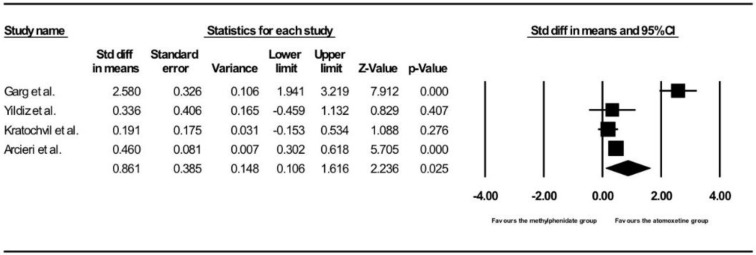
Forest plot of on post- vs. pre-treatment HR between children and adolescents receiving methylphenidate and atomoxetine treatment.

Figure 3 shows the results of the four studies that compared the post- vs. pre-treatment HR between children and adolescents taking methylphenidate and atomoxetine [13,43,44,45]. Children and adolescents treated with atomoxetine had a more significant increase in post- vs. pre-treatment HR than those treated with methylphenidate (pooled SMD with random-effects model: 0.86, 95% CI: 0.11–1.62, z = 2.24, *p* = 0.025). A significant level of between-study heterogeneity was found (τ^2^ = 0.52, Q = 44.19, df = 3, *p* <0.001, I^2^ = 93.21). When we undertook meta-regression to explore the impact of our a priori sources of heterogeneity (see Table 3), we found significant effects of mean age of participants (β = −0.079, z = −5.9, *p* < 0.0001), proportion of male gender (β = −17.7, z = −5.67, *p* < 0.001), mean dose of methylphenidate (β = −0.082, z = −5.12, *p* < 0.001), and mean dose of atomoxetine (β = −0.047, z = −5.27, *p* < 0.001). There was no publication bias (intercept = 2.79, SE = 3.75, df = 2, *p* = 0.53).

### 3.3. Systolic Blood Pressure

#### 3.3.1. Comparing Post- vs. Pre-Treatment SBP between the Methylphenidate and Placebo Groups (Children/Adolescents/Adults)

**Figure 4:**
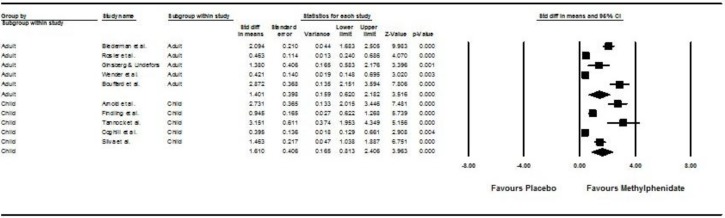
Forest plot and subgroup analysis on post- versus pre-treatment systolic blood pressure (SBP) between placebo and methylphenidate groups.

Figure 4 shows the results of the 10 studies that compared the post- vs. pre-treatment SBP between participants receiving methylphenidate and placebo treatment [32,34,35,37,38,39,40,41,42,46]. Children and adolescents treated with methylphenidate had a more significant increase in post- vs. pre-treatment SBP than those treated by placebo (pooled SMD with random-effects model: 1.61, 95% CI: 0.81–2.41, z = 3.96, *p* < 0.001). Adults treated with methylphenidate had a more significant increase in post- vs. pre-treatment SBP than those treated by placebo (pooled SMD with random-effects model: 1.40, 95% CI: 0.62–2.18, z = 3.52, *p* < 0.001). Subgroup analysis showed no significant difference between children/adolescents and adults in post- vs. pre-treatment SBP (Q = 0.14, *p* = 0.71). A significant level of between-study heterogeneity was found (τ^2^ = 0.54, Q = 134.19, df = 8, *p* < 0.001, I^2^ = 94.04). When we undertook meta-regression to explore the impact of our a priori sources of heterogeneity (see Table 4), we found significant effects of duration of treatment (B = −0.016, z = −3.07, *p* < 0.0001) and mean dose of methylphenidate (β = 0.013, z = 2.66, *p* = 0.0079). There was publication bias (intercept = 8.48, SE = 2.00, df = 7, *p* = 0.0039). The number of missing studies required to nullify results is 673 studies.

#### 3.3.2. Comparing Post- vs. Pre-Treatment SBP between the Methylphenidate and Atomoxetine Groups (Children/Adolescents Only)

**Figure 5:**
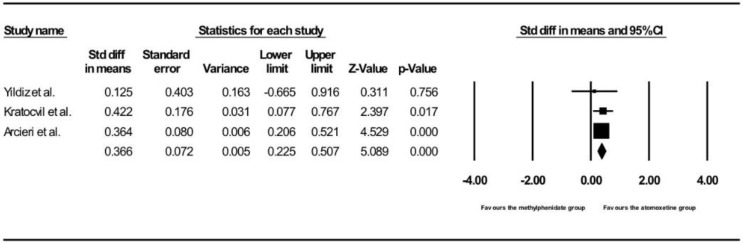
Forest plot of methylphenidate versus atomoxetine on post- vs. pre-treatment SBP in children and adolescents.

Figure 5 shows the results of the three studies that compared the post- vs. pre-treatment SBP between children and adolescents receiving methylphenidate and atomoxetine treatment [13,44,45]. Children and adolescents treated with atomoxetine had a more significant increase in post- vs. pre-treatment SBP as compared to those treated with methylphenidate (pooled SMD with random-effects model: 0.366, 95% CI: 0.23–0.51, z = 5.09, *p* < 0.001). No between-study heterogeneity was found (τ^2^ = 0, Q = 0.46, df = 2, *p* = 0.80, I^2^ = 0). Meta-regression was not performed because there was no between-study heterogeneity and because of the small number of studies that provided information on moderators. There was no publication bias (intercept = −0.38, SE = 0.65, df = 1, *p* = 0.67).

#### 3.3.3. Comparing Number of Adverse Cardiac Events between the Methylphenidate and Placebo Groups in Adults

**Figure 6:**
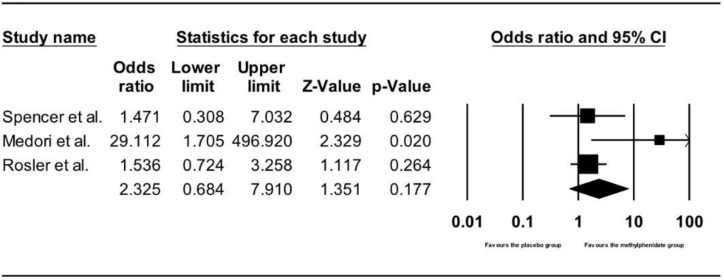
Forest plot on number of adverse cardiac events in adults receiving methylphenidate treatment and placebo.

Figure 6 shows the results of the three studies that compared the number of adverse cardiac events between adults receiving methylphenidate treatment and placebo [38,47,48]. There was no difference in the number of adverse cardiac events between the participants treated with methylphenidate and placebo (OR = 2.33, 95% CI: 0.68–7.91, z = 1.35, *p* = 0.18).

#### 3.3.4. Comparing Number of Adverse Cardiac Events between the Methylphenidate and Atomoxetine Groups in Children and Adolescents

**Figure 7:**
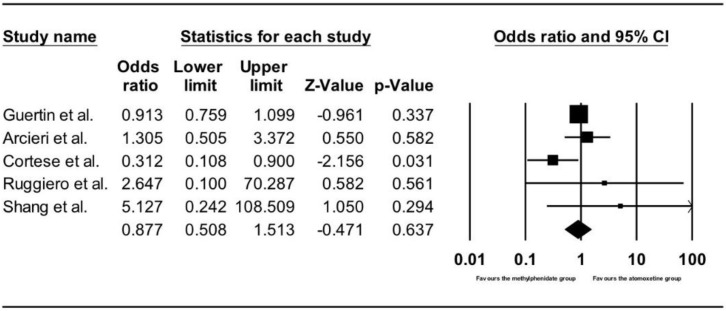
Forest plot on number of adverse cardiac events in children and adolescents receiving methylphenidate versus atomoxetine treatment.

Figure 7 shows the results of the five studies that compared the number of adverse cardiac events between children and adolescents receiving methylphenidate and atomoxetine treatment [13,49,50,51,52]. There was no difference in the number of adverse cardiac events between the participants treated with methylphenidate and atomoxetine (OR = 0.88, 95% CI: 0.51–1.51, z = −0.47, *p* = 0.64).

## 4. Discussion

### 4.1. Principal Findings

This meta-analysis found that children/adolescents and adults treated with methylphenidate resulted in significant increases in post- vs. pre-treatment HR and SBP as compared to placebo. Similarly, children and adolescents treated with atomoxetine had significant increases in post- vs. pre-treatment HR and SBP than those treated with methylphenidate. This meta-analysis is the first to perform a subgroup analysis that showed that children/adolescents and adults had similar risks in causing an increase in post- vs. pre-treatment HR and SBP after taking methylphenidate. We noticed a high level of heterogeneity that might be attributed to the differences in mean age of participants, mean doses, and duration of methylphenidate and atomoxetine treatment. In adults, there was no significant difference in the number of adverse cardiac events between participants treated with methylphenidate and placebo. In children/adolescents, there was no significant difference in the number of adverse cardiac events between participants treated with methylphenidate and atomoxetine.

### 4.2. Comparison with Other Studies

This meta-analysis found no statistically significant difference in the number of adverse cardiac events in adults receiving methylphenidate as compared to placebo. Our findings helped to address the mixed findings on the association between stimulant use and adverse cardiovascular outcomes reported by a systematic review in 2012 [14]. We further clarified these risks in children and adolescents taking methylphenidate and atomoxetine and found no significant difference in the number of adverse cardiac events between these two drugs.

This meta-analysis has confirmed that there were significant increases in post- vs. pre-treatment HR and SBP in children/adolescents and adults receiving methylphenidate or atomoxetine as compared to placebo, which is consistent with recent meta-analysis that reported similar findings in children and adolescents [17]. We further clarified these risks in children/adolescents and adults taking atomoxetine and found atomoxetine caused significant and larger increases in post- vs. pre-treatment HR and SBP as compared to methylphenidate.

Our findings challenged the conclusion from a previous review which stated that methylphenidate and atomoxetine caused small and nonsignificant increases in mean HR and BP in children and adolescents [15]. The aforementioned review was based on graphical and tabular summaries but not statistical analysis. Our findings support a previous meta-analysis that reported significant increases in resting mean HR and SBP associated with stimulant treatment in young adults [16]. Our conclusion is more robust because our analyses were based on a comparison between post- vs. pre-treatment HR and SBP rather than cross-sectional measurement of mean HR and SBP.

### 4.3. Mechanisms Leading to Increase in HR and SBP

Methylphenidate acts by inducing release of noradrenaline and dopamine into synaptic clefts and thus stimulating postsynaptic receptors [53] and stimulates the central nervous system. Atomoxetine is a selective noradrenaline reuptake inhibitor. The mechanisms leading to increase in SBP and HR in patients receiving atomoxetine and methylphenidate treatment is a topic of debate. Joyce et al. (1984) reported that methylphenidate caused an increase in plasma adrenaline, SBP, and HR without altering plasma noradrenaline [53]. Methylphenidate increases the mean HR by mediating the sympathetic [7], central, and peripheral catecholaminergic systems [54]. Wakamatsu et al. (2009) postulated that the increased plasma adrenaline levels were accompanied by central dopaminergic activation by methylphenidate [55].

For atomoxetine, Wakamatsu et al. (2009) suggested that atomoxetine increased BP and HR without affecting the plasma adrenaline concentration but the exact mechanism remains unknown [55]. Michelson et al. (2007) suggested that greater increases in cardiovascular tones in CYP 2D6 poor metabolizers for atomoxetine [56]. Kelly et al. (2005) found that acute dosing with atomoxetine increased both BP and HR on initial dosing, with lesser effects on HR and no effect on BP at day 5 as compared to methylphenidate [57]. In contrast, our meta-analysis found that atomoxetine caused significant changes in post- vs. pre-treatment HR and SBP in children and adolescents as compared to methylphenidate. This finding could not be explained by P450 2D6 poor metabolism. In other psychiatric conditions, increase in proinflammatory cytokines were found to be associated with increase in HR and SBP [58]. The relationship between atomoxetine and proinflammatory cytokines should be explored. Our finding provides a further research opportunity to elucidate how long-term atomoxetine treatment causes increases in HR and SBP.

### 4.4. Clinical Implications

This meta-analysis can potentially inform both clinicians and patients about the cardiovascular side effects associated with atomoxetine and methylphenidate to make an informed decision about its potential risks to increase HR and SBP. Our findings support the recommendations proposed by the Medicines and Healthcare products and Regulatory Agency (UK) [59]. It is important therefore that clinicians prescribing atomoxetine or methylphenidate should provide health education to patients as well as parents of children/adolescents to inform them about potential increase in HR and SBP. For contraindications, both atomoxetine and methylphenidate should not be used in children/adolescents and adults suffering from severe cardiovascular or cerebrovascular disorders. Atomoxetine and methylphenidate are contraindicated in patients for whom clinical deterioration would be expected, with increases in HR or SBP that could be clinically important (e.g., 20 beats per minute in HR and 15–20 mm Hg in SBP or 20 beats per minute in HR). For pre-treatment screening, children/adolescents and adults being considered for atomoxetine or methylphenidate treatment need a careful history and physical examination to assess any presence of cardiovascular disease or medical condition that can be worsened by increased HR and SBP. They should be referred for specialist cardiac evaluation if initial findings suggest such medical history or presence of cardiovascular disease. For monitoring, cardiovascular status should be regularly monitored before and during treatment, with BP and HR recorded appropriately after every dose adjustment and at least every six months to detect potentially clinically important increases. Hypertension and tachycardia caused by atomoxetine and methylphenidate should undergo a prompt specialist cardiac evaluation to consider antihypertensive and beta-blockers. Further studies are required to address whether supplementation with beta-blockers and antihypertensive medications are effective approach to prevent tachycardia and increase in SBP in patients receiving methylphenidate treatment.

### 4.5. Strengths and Limitations

We believe this is the first meta-analysis to study the effects of methylphenidate and atomoxetine on HR, SBP, and cardiac adverse events across all age groups including children/adolescents and adults. In this meta-analysis, 16 out of 22 studies (72.7%) achieved the Jadad score ≥3 that indicated good quality. Furthermore, 18 out of the 22 studies (81.8%) included are randomised controlled trials. In contrast, nearly 80% of studies included in a recent meta-analysis are open-label studies without a placebo group [17]. We performed meta-regression to identify moderators that explained heterogeneity. In contrast, a recent meta-analysis reported almost no effects of moderators [17].

There are a few limitations to our study. First, this meta-analysis mainly focused on SBP but not diastolic blood pressure (DBP). SBP has been a better predictor of risk than DBP [23]. Moreover, elevated SBP is the main target of antihypertensive therapy and isolated systolic hypertension predicts risk better than isolated diastolic hypertension [22]. Second, we did not evaluate the effects of other stimulants including amphetamine or lisdexamphetamine and alpha-adrenergic agonists, including clonidine and guanfacine, on BP and HR. Third, we did not have the information of CYP 2D6 metabolism of the participants and could not study the effect of CYP 2D6 metabolism on the cardiovascular tone in patients receiving atomoxetine treatment. Fourth, there were not enough studies comparing the effects on BP and HR between atomoxetine and placebo and thus further research is required.

## 5. Conclusions

Our meta-analysis demonstrated that children/adolescents and adults treated with methylphenidate resulted in significant increases in post- vs. pre-treatment HR and SBP as compared to placebo. Similarly, children and adolescents treated with atomoxetine had significant increases in post- vs. pre-treatment HR and SBP than those treated with methylphenidate. The results of this study can be applied by mental health professionals and paediatricians on patients who receive atomoxetine or methylphenidate treatment. Throughout the course of treatment, HR and SBP should be adequately monitored. Further research is required to elucidate how long-term atomoxetine treatment causes increases in HR and SBP. Patients and caregivers can be reassured that there was no significant difference in the number of adverse cardiac events in adults treated with methylphenidate and placebo as well as in children/adolescents treated with methylphenidate and atomoxetine.

## Figures and Tables

**Table 1:** Characteristics and quality of controlled trials comparing methylphenidate and either placebo or atomoxetine in patients with attention deficit hyperactive disorder.

Study	Study Design *	Comparison *	Dose	*n*	Age (Mean)	% Male	Study Population	Study Duration (Weeks)	Jadad Score
Arnold et al. (1978) [32]	RCT (CO)	MPH (*n* = 29) vs. placebo (*n* = 29)	MPH: 3.6 mg/day	58	8.0	0.759	Children	3	4
Kelly et al. (1988) [33]	RCT (CO)	MPH (*n* = 47) vs. placebo (*n* = 47)	MPH: 20 mg/week	94	8.3	0.936	Children	5	3
Findling et al. (2001) [34]	RCT	MPH (*n* = 82) vs. placebo (*n* = 82)	MPH: 5–15 mg/day	164	10.0	0.805	Children and adolescents	1	1
Biederman et al. (2006) [35]	RCT	MPH (*n* = 67) vs. placebo (*n* = 74)	MPH: 1.3 mg/kg/day	141	40.5	0.518	Adults	6	4
Barkley & Jackson (1977) [36]	RCT (CO)	MPH (*n* = 12) vs. placebo (*n* = 12)	MPH: 10–25 mg/day	24	8.2	1.000	Children	2	4
Tannock et al. (1989) [37]	RCT (CO)	MPH (*n* = 12) vs. placebo (*n* = 12)	MPH: 1 mg/kg/day	24	8.4	0.830	Children	0.86	4
Rosler et al. (2009) [38]	RCT	MPH (*n* = 241) vs. placebo (*n* = 118)	MPH: 10–60 mg/day	359	34.7	0.496	Adults	24	3
Ginsberg & Lindefors (2011) [39]	RCT	MPH (*n* = 15) vs. placebo (*n* = 15)	MPH: 36–72 mg/day	30	34.4	1.000	Adults	47	5
Bouffard et al. (2003) [40]	RCT (CO)	MPH (*n* = 30) vs. placebo (*n* = 30)	MPH: 30–45 mg/day	60	34.0	0.800	Adults	4	5
Coghill et al. (2013) [41]	RCT	MPH (*n* = 111) vs. placebo (*n* = 110)	MPH: 18–54 mg/day	221	10.9	0.819	Children and adolescents	7	5
Silva et al. (2005) [42]	RCT (CO)	MPH (*n* = 54) vs. placebo (*n* = 54)	MPH: 18–40 mg/day	108	9.4	0.630	Children	6	3
Garg et al. (2014) [43]	RCT	MPH (*n* = 33) vs. ATX (*n* = 36)	MPH: 0.2–1 mg/kg/day ATX: 0.5 mg/kg/day	69	8.6	0.812	Children	8	3
Yildiz et al. (2011) [44]	RCT	MPH (*n* = 11) vs. ATX (*n* = 14)	MPH: 18–54 mg/day ATX: 0.5–1.2 mg/kg/day	25	9.9	0.880	Children	12	2
Kratochvil et al. (2002) [45]	RCT	MPH (*n* = 40) vs. ATX (*n* = 180)	MPH: 15–60 mg/day ATX: 0.2–1 mg/kg/day	220	10.4	0.925	Children and adolescents	10	3
Wender et al. (2011) [46]	RCT (CO)	MPH (*n* = 105) vs. placebo (*n* = 105)	MPH: 30–60 mg/day	210	36.9	0.724	Adults	52	5
Spencer et al. (2007) [47]	RCT	MPH (*n* = 165) vs. placebo (*n* = 53)	MPH: 20–40 mg/day	218	38.6	0.574	Adults	5	3
Medori et al. (2008) [48]	RCT	MPH (*n* = 102) vs. placebo (*n* = 96)	MPH: 18–72 mg/day	198	34.0	0.576	Adults	5	4
Arcieri et al. (2012) [13]	CS	MPH (*n* = 315) vs. ATX (*n* = 316)	MPH: 0.3–0.6 mg/kg/day ATX: 0.5–1.2 mg/kg/day	631	10.6	0.883	Children and adolescents	52	NA
Guertin et al. (2014) [49]	CS	MPH (*n* = 37011) vs. ATX (*n* = 3595)	Variable doses	40606	9.1	0.703	Children	13	NA
Cortese et al. (2015) [50]	Retrospective CS	MPH (*n* = 1426) vs. ATX (*n* = 985)	MPH: 0.3–0.6 mg/kg/day ATX: 0.5–1.2 mg/kg/day	2411	10.7	0.881	Children and adolescents	240	NA
Ruggiero et al. (2012) [51]	Retrospective CS	MPH (*n* = 8) vs. ATX (*n* = 68)	Variable doses	76	9.6	0.868	Children	Variable duration	NA
Shang et al. (2015) [52]	RCT	MPH (*n* = 80) vs. ATX (*n* = 80)	MPH: 18–54 mg/day ATX: 0.5–1.2 mg/kg/day	160	9.8	0.875	Children and adolescents	24	3

* Legend: RCT-randomized controlled trials; CO-crossover study; CS-cohort study; MPH-methylphenidate; ATX-atomoxetine.

**Table 2:** Meta-regression analysis that explored the source of heterogeneity on the post- versus pre-treatment HR between placebo and methylphenidate groups.

Moderators	No. of Studies Used	Slope	Standard Error	Lower Limit (95% CI)	Upper Limit (95% CI)	Z-Value	*p*-Value
Mean age of all participants	11	0.0032	0.00044	0.0023	0.0041	7.31	<0.001
Proportion of male gender in all participants	11	−1.88	0.41	−2.70	−1.06	−4.50	<0.001
Duration of treatment of the methylphenidate and placebo groups	11	0.011	0.0055	0.00057	0.022	2.06	0.04
Mean dose of methylphenidate	10	0.032	0.0049	0.022	0.042	6.53	<0.001

**Table 3:** Meta-regression analysis that explored the source of heterogeneity on post- versus pre-treatment HR between children and adolescents receiving methylphenidate and atomoxetine treatment.

Moderators	No. of Studies Used	Slope	Standard Error	Lower Limit (95% CI)	Upper Limit (95% CI)	Z-Value	*p*-Value
Mean age of all participants	4	−0.079	0.013	−0.10	−0.05	−5.90	*p* < 0.001
Proportion of male gender in all participants	4	−17.7	3.12	−23.82	−11.59	−5.67	*p* < 0.001
Duration of treatment of the methylphenidate and atomoxetine groups	4	−0.02	0.01	−0.04	0.002	−1.77	0.08
Mean dose of methylphenidate	4	−0.082	0.02	−0.11	−0.05	−5.12	*p* < 0.001
Mean dose of atomoxetine	4	−0.047	0.009	−0.065	−0.03	−5.27	*p* < 0.001

**Table 4:** Meta-regression analysis that explored the source of heterogeneity on the post- vs. pre-treatment SBP between placebo and methylphenidate groups.

Moderators	No. of Studies Used	Slope	Standard Error	Lower Limit (95% CI)	Upper Limit (95% CI)	Z-Value	*p*-Value
Mean age of all participants	10	−0.00037	0.00038	−0.0011	0.00037	−0.98	0.33
Proportion of male gender in all participants	10	0.19	0.42	−0.63	1.00	0.44	0.65
Duration of treatment of the methylphenidate and placebo	10	−0.016	0.0053	−0.026	−0.0058	−3.07	0.0022 *
Mean dose of methylphenidate	9	0.013	0.0050	0.0035	0.0023	2.66	0.0079 *

* *p* < 0.050
